# Assessment of Microplastic Exposure in Diabetic Patients Using Insulin

**DOI:** 10.3390/toxics13110926

**Published:** 2025-10-29

**Authors:** Duygu Felek, Mustafa Fatih Erkoc, Merve Yaylacı, Vugar Ali Turksoy

**Affiliations:** 1 Department of Internal Medicine, Faculty of Medicine, Yozgat Bozok University, Yozgat 66100, Türkiye; 2 Department of Radiology, Faculty of Medicine, Yozgat Bozok University, Yozgat 66100, Türkiye; mustafafatih.erkoc@bozok.edu.tr; 3 Department of Medical Biochemistry, Yozgat Sorgun State Hospital, Yozgat 66100, Türkiye; 4 Department of Public Health, Faculty of Medicine, Yozgat Bozok University, Yozgat 66100, Türkiye

**Keywords:** diabetes mellitus, insulin, microplastics

## Abstract

This study investigates the potential role of microplastics in the development of diabetes mellitus and assesses their presence in individuals undergoing insulin therapy. A total of 100 participants were included: 50 insulin-dependent diabetic patients and 50 healthy controls. The diabetic group was divided into two subgroups based on their insulin regimen: those receiving one daily injection of basal insulin and those receiving four injections of basal and short-acting insulin. Blood samples were analysed for microplastic content using chromatographic methods (LC/GC-MSMS and LCTOF MS). The findings revealed that diabetic patients had significantly higher serum microplastic levels (3.14 ± 1.30 µg/mL) than healthy individuals (1.50 ± 0.89 µg/mL, *p* < 0.05). Within the diabetic group, patients receiving four injections had a longer disease duration (15.14 ± 3.64 years) than those receiving one injection (10.56 ± 5.21 years), with a statistically significant difference (*p* = 0.001). However, microplastic levels did not differ significantly based on injection frequency. A strong positive correlation was observed between microplastic levels and both HbA1c (%) and fasting glucose levels (*p* = 0.001). These results imply that microplastics may act as endocrine disruptors that contribute to the development of diabetes, rather than being introduced through insulin treatment itself.

## 1. Introduction

The increasing use of plastic endangers both our ecosystem and our health. As a result of its uncontrolled use, only a small proportion of plastic waste can be recycled, with the majority accumulating in the environment where it can remain for many years without decomposing [1,2]. Exposure to plastic-derived particles is ubiquitous. Microplastics enter the food chain due to their persistence in the environment and ultimately reach humans through multiple pathways. A prime example of this is the presence of microplastics in ocean waters and on uninhabited islands, where no pollutants have been found [3]. The levels of microplastics to which we are exposed through drinking water are quite alarming [4]. These ingested plastic particles cause a number of pathologies in the body. Studies on humans and animals indicate that the gastrointestinal system is most affected, and exposure throughout life, including in the womb, demonstrates the extent to which our health is threatened [5,6]. The literature also suggests that microplastics accumulate in the body over time, resulting in long-term effects [7]. There is strong evidence that microplastics play a role in the aetiology of certain endocrine disorders. Animal modelling studies, specifically rat experiments involving oral exposure to microplastic-containing materials, have highlighted the role of micro- and nanoplastics in the aetiology of diabetes mellitus, drawing attention to metabolic outcomes. It provides a theoretical framework and external data to support the biological plausibility (i.e., mechanisms) and/or observational links between plastics and diabetes risk, in order to inform human clinical trials [8,9]. The relationship between the intake of food containing microplastics and obesity has been discussed, along with the possibility that this may lead to endocrinopathies [10]. Furthermore, as plastic exposure is also associated with chronic diseases, a link with diabetes mellitus is suggested, which is quite common in our country as well as worldwide [11,12].

Microplastic exposure can occur iatrogenically, as well as through physiological nutrition and contact. Microplastic exposure through the use of medical devices encompasses a wide range of factors, from the materials used in treatment to the disposal of medical waste and its subsequent entry into the natural environment [13]. An example of plastic content in medical materials is haemodialysis fluids, which are well documented in the literature as a source of exposure to medical materials [14]. It has been reported that the use of medical supplies, particularly for intravenous applications, increases microplastic exposure [15]. While most studies focus on the environmental risks of medical waste, our study aims to raise awareness of the microplastics we are exposed to through medical supplies and propose measures to address this issue. Our study will examine plastic exposure during the stages leading up to the administration of daily insulin, as well as the microplastic exposure of patients using insulin. Considering that 11.12% of people in Turkey have diabetes, we believe that this is an issue that needs to be evaluated on a global scale, and that applying safe, reversible treatment that minimises harm would benefit patients [16]. Our study will evaluate the role of microplastics in the aetiology of diabetes mellitus, as well as the association between microplastic exposure and insulin pen use. The study is expected to contribute to the existing literature in multiple ways. In light of the lack of clinical studies on exposure among diabetic patients, and bearing in mind the size of the global diabetic patient population and the amount of medical waste generated after insulin administration, we believe this study is necessary to protect individuals and the environment.

## 2. Materials and Methods

### 2.1. Design and Participants

The study included 50 patients diagnosed with type 2 diabetes mellitus who were using insulin and attended the Internal Medicine Clinic at Yozgat Bozok University, as well as 50 healthy control subjects. The Turkish Endocrinology and Metabolism Association’s Diabetes Mellitus Diagnosis and Treatment Guide was used as the diagnostic criterion for diabetes mellitus [17]. The study included individuals aged 18 years and over who had been followed up for at least one year for diabetes mellitus and had been using subcutaneous insulin for at least six months. The healthy control group consisted of individuals who visited the same clinic and were found to be healthy based on physical examination and tests. As patients with type 1 diabetes mellitus, which is prevalent in young age groups, were not included in our study, the patient group consisted of individuals with type 2 diabetes mellitus in older age groups, which is consistent with the literature. Thus, autoimmunity due to type 1 diabetes mellitus was excluded. Since the participants live in the same region, exposure through inhalation is less likely, but local nutritional habits are similar, so the effect of environmental factors on exposure in our study was limited.

### 2.2. Data Collection and Analysis

All venous blood samples were collected between 08:00 and 10:00 a.m., after a fasting period of at least 8 h, to minimise metabolic variation. The samples were obtained using metallic needles and glass syringes, then transferred to polymer-free biochemistry tubes. The samples were then centrifuged within 15 min of collection at 3000 rpm. The serum was separated and stored in glass Eppendorf tubes at −80 °C until analysis. All experimental procedures were conducted in a Class II laminar flow cabinet, and all laboratory surfaces were thoroughly cleaned with 70% ethanol prior to each run. To estimate background contamination, environmental blanks (open glass tubes exposed during sampling) and procedural blanks (reagents processed without biological samples) were analysed simultaneously with each batch. The mean microplastic levels detected in the blanks were below the limit of detection (LOD = 0.05 µg/mL), indicating negligible contamination. Multi-polymer reference standards (polyethylene, polypropylene, polyvinyl chloride and polyethylene terephthalate, from Sigma Aldrich) were used for calibration at concentrations of 0.1–10 µg/mL. Calibration curves for each polymer type showed excellent linearity (R^2^ ≥ 0.995). Daily calibration verification was performed using mid-level standards (2 µg/mL). During the study, microplastic levels from biological tubes and related equipment were analysed five times a day (within and between days) for ten days; these levels were considered blanks. Furthermore, all processes (extraction, PDA, HPLC and GC-MS) other than those involving blood tubes were performed using metallic and glass materials. Therefore, contamination from microplastics was prevented. Preliminary analysis and extraction were performed at the Multidisciplinary Research Laboratory of Yozgat Bozok University, after which the collected samples were analysed for the presence of microplastics. Several methods were employed to determine the presence of microplastics, in order to detect plastic particles of various properties and sizes. For the study, 100 µL of each blood sample was extracted using organic solvents under the aforementioned acidity/alkalinity conditions and then analysed at specific wavelengths using a photodiode array (PDA) detector. The tubes were tightly closed and shaken thoroughly for 90 min in an ultrasonic bath at 40 °C. They were then centrifuged at 14,000 rpm for 10 min. 500 µL of the supernatant was transferred to an HPLC vial and 20 µL of this was injected into the HPLC system. Microplastic levels were determined in the study. The study used an InertsilODS-4 C18 column (5 µm particle size, 4.6 mm × 250 mm). Based on the studies conducted, the most suitable procedure for the linear gradient was determined to be the use of the mobile phase (acetonitrile and ultra-pure water). A linear gradient was performed over 28 min, starting at 0% acetonitrile and ending at 100% acetonitrile. Gradient elution was applied to separate the solvents simultaneously at 5.00 min with 0% acetonitrile. This was then increased to 50% acetonitrile at 5.01 min, which was held until 9.00 min. This was then increased to 70% acetonitrile at 9.01 min, which was held until 15.00 min. It was then increased to 100% acetonitrile at 15.01 min, which was held until 28.00 min. The system was then gradually returned to the initial gradient and flow rate for the next sample (two minutes). To shorten the run time and sharpen the peaks, the flow rate was increased to 1.5 mL/min from 22.00 to 24.00 min, and then maintained until 28.00 min. It was then decreased to 1.0 mL/min within 1.0 min. GC-MS analysis was performed to analyse the solvent. For this method, a 5 ms silica capillary column (30 m × 0.25 mm × 0.25 mm) was used, and the relevant method parameters were applied. Injector temperature: 290 °C; split ratio: 1:20; detector temperature: 320 °C.

Quadrupole: 150 °C; injection volume: 1.0 µL; oven temperature: set to 70 °C for 1 min, then increased by 10 °C per minute up to 280 °C. Microplastics were detected [18,19]. The limit of detection (LOD) of the analytical method was determined as 0.05 µg/mL, based on a signal-to-noise ratio of 3:1. The limit of quantification (LOQ) was 0.15 µg/mL (S/N = 10:1). Recovery was assessed by spiking blank serum samples with microplastic (MP) standards at concentrations of 1, 3 and 5 µg/mL. This yielded recovery rates ranging from 92.3% to 104.5%, with inter-day precision below 3.21% (RSD).

### 2.3. Exclusion Criteria

Separate exclusion criteria were established for the patient and healthy groups. In the patient group, individuals with any chronic condition other than diabetes mellitus were excluded from the study. Patients with type 1 diabetes mellitus, which includes autoimmunity factors, were also excluded. Individuals using oral antidiabetic agents or undergoing continuous medical treatment (except insulin use) were also excluded. In the healthy group, individuals using long-term medical agents or complementary medicine products were excluded from the study, even if they did not have a chronic disease. In both groups, individuals with foreign bodies containing plastic and individuals who smoked or consumed alcohol were excluded from the study.

### 2.4. Statistical Analysis

The data distribution was assessed using the Shapiro–Wilk test for n < 200 and was visually confirmed using Q–Q plots and histograms. Variables showing a normal distribution were compared using an independent samples *t*-test or one-way ANOVA, while variables that were not normally distributed were analysed using a Mann–Whitney U test or a Kruskal–Wallis test. Spearman’s rank correlation coefficient was used to evaluate correlations for non-parametric data. All statistical analyses were performed using SPSS 20.0 (Chicago, IL, USA). A two-tailed significance level of *p* < 0.05 was considered statistically significant, whereas *p* < 0.01 indicated a highly significant result. In addition to *p*-values, 95% confidence intervals (CIs) and effect size measures were reported to evaluate the strength of the association. Cohen’s d was used to compare continuous variables, and odds ratios (OR) with 95% confidence intervals (CI) were calculated for categorical outcomes. For regression models, standardised β coefficients with their respective 95% CIs were presented. Multivariate linear regression analyses were performed to account for potential confounders. Serum microplastic level (µg/mL) was considered the dependent variable, while diabetes status, age, sex and BMI were included as independent variables. Variance inflation factors (VIF < 2) confirmed the absence of multicollinearity.

## 3. Results

The study included 100 individuals, comprising 50 patients and 50 healthy controls. Of the patient group, 60% were female and 40% were male, while of the control group, 72% were female and 28% were male. No statistically significant difference in gender distribution was found between the two groups (*p* = 0.209). The mean age of the patients included in the study was 62.46 ± 9.62, compared to 41.76 ± 10.76 for the healthy controls. The patient group was older, and a statistically significant difference in age was found between the groups (*p* = 0.001).

**Figure 1:**
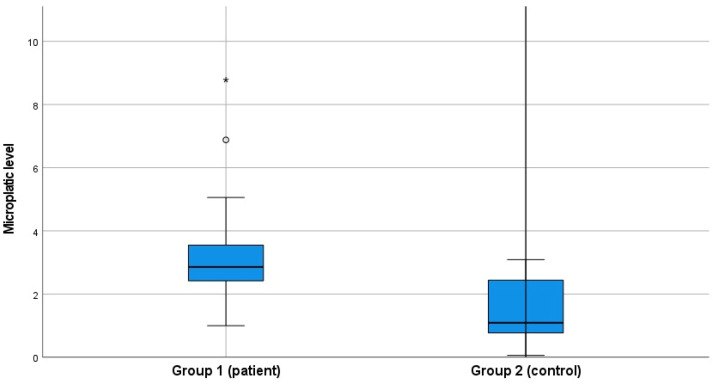
Microplastic levels between groups.

The groups were defined as having or not having diabetes mellitus. In the patient group, the mean HbA1c was 11.02% ± 1.15%, and the mean fasting blood glucose level was 248.36 mg/dL ± 85.12 mg/dL. In the control group, the mean HbA1c was 5.49% ± 0.41%, and the mean fasting blood glucose level was 91.60 mg/dL ± 7.18 mg/dL. There was a statistically significant difference between the groups in terms of mean HbA1c and glucose levels (*p* < 0.001). When microplastic levels were evaluated between the two groups, the mean microplastic level was found to be 3.14 ± 1.30 µg/mL in the patient group and 1.50 ± 0.89 µg/mL in the healthy control group. A statistically significant difference was found between the two groups in terms of microplastic levels (Table 1, Figure 1).

The correlation between the age, blood sugar levels and disease duration of the patients included in the study, and their microplastic levels, was examined. A positive correlation was observed between age, HbA1c%, fasting blood glucose level, diabetes mellitus duration, and microplastic levels (* *p* = 0.05, ** *p* = 0.01). Similarly, a positive correlation was observed between high blood sugar levels and microplastic levels (** *p* = 0.01). However, no correlation was observed between disease duration and microplastic presence (*p* > 0.05) (Table 2).

The patient group had insulin-dependent diabetes mellitus and was categorised based on the number of injections received. Among patients receiving a single injection, the mean HbA1c level was 11.17% ± 1.26%, and the mean fasting blood glucose level was 252.19 mg/dL ± 83.14 mg/dL. The group that received four insulin injections had an average HbA1c level of 10.95% and an average fasting blood glucose level of 232.12 mg/dL. When the duration of diabetes mellitus was compared among patients grouped by the number of injections received, the average duration was found to be 10.56 ± 5.21 years for patients receiving a single injection, and 15.14 ± 3.64 years for patients receiving four injections. This difference was statistically significant (*p* = 0.001). However, no difference was found between the groups in terms of mean HbA1c and glucose levels (*p* = 0.544 and 0.468, respectively). When microplastic levels were evaluated between the groups, the mean microplastic level was found to be 3.01 ± 0.89 µg/mL in the group receiving a single injection and 3.21 ± 1.47 µg/mL in the group receiving four injections. No statistically significant difference was found between the groups in terms of microplastic level (*p* = 0.624) (Table 3).

In the subgroup analysis of patients with diabetes, those receiving four daily insulin injections had a longer disease duration and similar HbA1c and fasting glucose levels compared with those on a single basal regimen. These comparisons are summarised in Table 3. Our results demonstrate elevated serum microplastic concentrations in diabetic patients (mean 3.14 ± 1.30 µg/mL) compared with healthy controls (mean 1.50 ± 0.89 µg/mL). This finding is consistent with those of Leslie et al. [20] (mean 1.60 µg/mL) and Brits et al. [21] (range 0.17–2.49 µg/mL). Similar exposure magnitudes were also reported by Dong et al. [22] at a mean of 2.46 µg/mL, which supports the reliability of our analytical findings [23]. Rather than restating tabulated data, our discussion now emphasises these cross-study comparisons and their mechanistic implications (Table 4).

Multiple linear regression analysis, adjusted for age, sex, disease duration, BMI, smoking status, insulin therapy and socioeconomic characteristics, revealed that HbA1c and fasting glucose levels were independently associated with serum microplastic concentrations (*p* < 0.01). This finding suggests that poor glycaemic control could contribute to microplastic accumulation, regardless of demographic or lifestyle factors (see Table 5).

Regression analyses were performed using two models. In Model A, the dependent variable was microplastic level (µg/mL), and the independent variables were diabetes status, age, sex, BMI and smoking. In Model B, HbA1c (%) was the dependent variable and the independent variables were microplastic level, age, sex, disease duration and BMI. Both models were adjusted for potential confounders, and the results are presented as standardised β coefficients with 95% confidence intervals. A multivariate regression model was constructed to evaluate independent predictors of serum microplastic levels, adjusting for age, sex, and BMI. Diabetes status (β = 0.486, *p* < 0.001) and age (β = 0.241, *p* = 0.022) were found to be significant predictors, whereas sex and BMI were not significant (*p* > 0.05). The model explained approximately 43% of the variance (adjusted R^2^ = 0.43). These findings suggest that, while microplastic accumulation increases with age, diabetes contributes independently to higher microplastic levels.

## 4. Discussion

The mean age of the patients included in the study was 62.46 ± 9.62 years; for the healthy group, it was 41.76 ± 10.76 years. The difference between the two groups was found to be statistically significant. As the healthy group consisted of patients who visited the internal medicine clinic without any disease, a lower result was expected. A foreign study involving 19,327 patients with type 2 diabetes mellitus determined that the average age of disease onset was 60.2 years [24]. A field study conducted in Turkey found that the average age of individuals diagnosed with diabetes was approximately 51.1 ± 11.4 years, with a slightly higher prevalence of type 2 diabetes in women [25]. Considering the age of onset of chronic diseases, our study’s findings are consistent with those in the literature.

When we analysed the microplastic levels of the individuals included in the study, we found that the average level was 3.14 ± 1.30 µg/mL in the patient group and 1.50 ± 0.89 µg/mL in the healthy control group. The difference between the two groups was significant (*p* = 0.001). This made us wonder whether microplastics play a role in the aetiology of diabetes mellitus. A study conducted in Australia analysed measurable plastic particles in the urine of men and discussed their correlation with existing chronic diseases. Ultimately, plastic content was found to be a risk factor for cardiovascular disease, diabetes and hypertension (*p* = 0.001, 0.001 and 0.013, respectively), but no correlation was found with asthma or depression diagnoses [26]. Another study, this time in Korea, examined the role of plastic particles in the aetiology of obesity and diabetes mellitus, detecting high levels of these chemical contaminants in the urine of patients in these groups [27]. Similarly, Hsiao et al. highlight plastic pollutants as a risk factor for diabetes mellitus in their study, which supports our own findings by showing the positive relationship between HbA1c/glucose and MP levels [8]. Literature reviews have shown that microplastic content plays a role in the aetiology of non-communicable chronic diseases. Considering that exposure to microplastics, which has been linked to the development of diabetes, was also found to be high in the patient group in our study, the results of the study conducted by Xu and colleagues are almost identical to those of our own study. This study, which was conducted on animal subjects, also shows that exposure to microplastics affects the gut microbiota, leading to induced glucose metabolism disorder in rats fed a high-fat diet and creating a predisposition to diabetes mellitus. Furthermore, the study addressed the cellular effects of plastic and glucose toxicity in relation to glucose levels and microplastic exposure [12]. Another study on diabetic rats found that the effects of plastics on the gut microbiota were accompanied by lung damage caused by the simultaneous inhalation of pollutants. This emphasises the need to protect against the undesirable effects of microplastics as environmental pollutants in the aetiology, management and treatment of diabetes mellitus [28]. A study on fish larvae was included in the literature as an in vivo and in vitro model, proving that microplastics cause disturbances in insulin production (INS alpha) and fat metabolism and suppress systemic inflammatory cytokine levels. This leads to metabolic problems, such as type 2 diabetes [29]. A methodological study presents data indicating an association between diabetes mellitus and micro- and nanoplastics; however, their role in human health remains to be discovered [30]. What sets our study apart is that it does not rely on animal models but instead measures plastic levels directly in individuals with diabetes mellitus. In our study, the mean HbA1c level in the patient group was 11.02% ± 1.15%, and the mean fasting blood glucose level was 248.36 mg/dL ± 85.12 mg/dL. In the healthy group, the mean HbA1c was 5.49% ± 0.41 and the fasting blood glucose level was 91.60 mg/dL ± 7.18. Despite receiving insulin treatment, the minimum HbA1c value of 9.6 mg/dL indicates that not all participants are within the target range according to the reference values in the Turkish Endocrinology and Metabolism Guidelines Diabetes Mellitus Diagnosis and Treatment Guide [17]. These results suggest that if microplastics are among the etiological factors of diabetes, regulation may be difficult or impaired. It could even be interpreted that exposure to microplastics negatively affects the management of diabetes mellitus and may complicate glycaemic control. Another piece of data supporting this view is that the patient group had insulin-dependent diabetes mellitus. When the patients were grouped and analysed according to the number of subcutaneous injections they performed, it was found that the average disease duration since diagnosis was 10.56 ± 5.21 years for those who performed a single injection, while it was 15.14 ± 3.64 years for patients who administered four injections. The difference between the groups was statistically significant (*p* = 0.001). This is an expected result, as the pancreatic insulin reserve gradually decreases during diabetes mellitus treatment, potentially increasing the patient’s insulin requirements. However, the results show that patients were not regulated, even when using single or quadruple insulin regimens. These findings suggest that exposure to microplastics may negatively impact glycaemic regulation. Although microplastic levels did not differ significantly by disease duration or treatment intensity, overall concentrations were higher in diabetic patients than in healthy controls. Microplastic levels, independent of disease duration and treatment, were found to be a risk factor for the development of the disease and treatment resistance after disease onset. No such study has been found in the literature. Examining the correlation between the patients’ age, blood sugar levels and disease duration and their microplastic levels revealed a positive relationship between age, HbA1c, fasting blood glucose level, duration of diabetes mellitus and microplastic levels (*p* = 0.001, 0.001, 0.018, 0.001). This is an expected result, consistent with data from statistical agencies, given that diabetes prevalence increases with age and the number of people with diabetes among the elderly is projected to reach alarming levels. According to data from the International Diabetes Federation (IDF), 537 million elderly people were monitored for diabetes in 2021, and this figure is expected to rise to 643 million by 2030 and 738 million by 2045 [31]. The positive correlation observed between high blood sugar and microplastic levels suggests that exposure to microplastics may cause poor glycaemic control, or alternatively, that microplastic exposure may be involved in the aetiology of poor glycaemic control (*p* = 0.001 for HbA1c; *p* = 0.001 for fasting blood glucose). A study by Zeng et al. indirectly explains the relationship between poor glycaemic control and diabetes. The risk of diabetic nephropathy developing as a result of uncontrolled diabetes treatment is a striking example in the literature, showing us the scale of the problem if no action is taken [32]. Some medical supplies are made of plastic and are designed for single use, which poses a threat to both the ecosystem and our health. In particular, injectable supplies have been identified as a risk factor. In a study by Chen et al., the microplastic content of infusion tubes and blood needles was measured using a spectrometric method. Exposure to polyamide, polyvinyl chloride, polyethylene and polyurethane was demonstrated. The researchers recommended taking precautions against toxicity from these plastic contents [33]. Kim et al., on the other hand, addressed microplastic exposure in relation to medical supplies such as disposable masks [34]. A study examining dialysis fluids in haemodialysis patients, where prolonged contact with blood is thought to be most intense, has also been documented in the literature as an example of medical exposure. In our study, the patient group consisted of patients with insulin-dependent diabetes mellitus; they were grouped and analysed according to the number of subcutaneous injections they performed, and we evaluated whether repeated contact with plastic medical supplies was a risk factor. The average microplastic level was calculated as 3.01 ± 0.89 µg/mL in the group performing a single injection and 3.21 ± 1.47 µg/mL in the healthy control group. No difference was found between the groups in terms of microplastic level. Unlike studies conducted with different materials, this result showed us that the storage and administration conditions of insulin do not cause microplastic exposure, making it a safe method in terms of microplastic content. However, it is a proven research finding that insulin pens contain polypropylene plastic material [15]. Therefore, while it does not pose a microplastic threat through application, it does not negate the fact that it is a pollutant posing an environmental threat [35]. The study by Casell et al. showed that medical devices (e.g., infusion sets, haemodialysis needles and tubes) can be a detectable source of microplastics (MP) but concluded that the higher burden in patients with type 2 diabetes cannot be explained solely by the iatrogenic effects of pens/insulin. Furthermore, the lack of a relationship between the number of insulin injections and microplastic exposure in our study provides additional support for this result in the existing literature [36]. After adjusting for age, sex and BMI, diabetes remained an independent predictor of serum microplastic concentration. Although a moderate correlation between age and microplastic levels was confirmed, the persistence of a strong association between diabetes and microplastics suggests that metabolic or treatment-related mechanisms may also contribute to microplastic accumulation beyond that related to age.

The number of participants in the study is one factor that can be increased, and this is the first limitation of our study. The second limitation is that, while the total microplastic load was measured, its subtypes (diethyl phthalate (DEP), diallyl phthalate (DAP), benzyl butyl phthalate (BBP), di-n-butyl phthalate (DBP), dihexyl phthalate (DHP), dicyclohexyl phthalate (DCHP), di-2-ethylhexyl phthalate (DEHP), di-n-octyl phthalate (DOP), diisononyl phthalate (DINP) and diisodecyl phthalate (DIDP)) were not.

In conclusion, the higher microplastic levels found in individuals diagnosed with diabetes mellitus, compared to the healthy group, suggest a level of microplastic effect on the aetiology of the disease. Although the microplastic ratio was higher in individuals who had received many injections, the difference was not significant. This result reinforced the conclusion that microplastic levels are involved in the aetiology of diabetes mellitus as endocrine disruptors rather than being iatrogenic increases due to insulin use.

## Figures and Tables

**Table 1:** Biochemical data and microplastic levels between groups.

	Patient (n = 50)	Control (n = 50)	Cohen’s d	95% CI for d	*p*
**HbA1c (%)**	11.02 ± 1.15	5.49 ± 0.41	4.68	[3.95–5.41]	<0.001
**Glucose (mg/dL)**	248.36 ± 85.12	91.6 ± 7.18	2.48	[2.01–2.95]	<0.001
**Microplastic levels (µg/mL)**	3.14 ± 1.30	1.50 ± 0.89	1.38	[1.01–1.75]	<0.001

**Table 2:** Correlation between microplastic levels and general data and laboratory parameters.

	HbA1C	Glucose	Time of Illness	MP
**Age**	0.682 **	0.634 **	0.334 *	0.528 **
**HbA1C (%)**	1	0.761 **	0.091	0.569 **
**Glucose (mg/dL)**		1	−0.007	0.484 **
**Time of illness**			1	−0.005
**Microplastic levels**				1

(* *p* < 0.05; ** *p* < 0.01).

**Table 3:** Biochemical data and microplastic levels according to the number of insulin injections in the patient group.

	Single Injection [n = 16 (%32)]				Four Injection [n = 34 (%68)]				
	Mean	STD	Min.	Max.	Mean	STD	Min.	Max.	*p*
**HbA1C (%)**	11.17	1.26	9.50	13.10	10.95	1.12	9.40	14.60	0.544
**Glucose (mg/dL)**	252.19	83.14	74.00	404.00	232.12	93.54	74.00	479.00	0.468
**Time of illness**	10.56	5.21	2.00	17.00	15.15	3.64	5.00	19.00	0.001 *
**Microplastic levels (µg/mL)**	3.01	0.89	1.93	5.06	3.21	1.47	1.00	8.78	0.624

* *p* < 0.01.

**Table 4:** Literature comparisons on human blood microplastic levels.

Studies	n	Measurement
Leslie et al., 2022 [20]	22	1.60 µg/mL
Dong et al., 2024 [22]	20	2.46 µg/mL (1.84–4.65 µg/mL)
Brits et al., 2024 [21]	68	1.07 µg/mL (0.17–2.49 µg/mL)
Leonard et al., 2024 [23]	20	2.466 ± 4.174 MP/mL
Our study (diabetic patients)	50	3.14 ± 1.30 µg/mL
Our study (healthy individuals)	50	1.50 ± 0.89 µg/mL

**Table 5:** Multiple Linear Regression Analysis for Predictors of Serum Microplastic Levels in Diabetic Patients.

Variable	β (Standardised Coefficient)	t	*p*-Value
**Age (years)**	0.112	1.47	0.145
**Sex (Female = 1, Male = 0)**	0.081	0.99	0.324
**Duration of disease (years)**	0.121	1.39	0.168
**BMI (kg/m^2^)**	0.095	1.21	0.229
**Smoking status (Yes = 1)**	0.072	0.87	0.388
**Insulin therapy (4 vs. 1 injection)**	0.043	0.52	0.604
**HbA1c (%)**	0.412	4.76	**<0.001** *
**Fasting glucose (mg/dL)**	0.297	2.85	**0.006** *
**Socioeconomic status**	0.062	0.81	0.418

Model R^2^ = 0.46; Adjusted R^2^ = 0.42; Model Significance (*p*) < 0.001. * *p* < 0.05.

## Data Availability

The original contributions presented in this study are included in the Supplementary Materials. The data that support the findings of this study are available from the corresponding author upon reasonable request.

## Supplementary Materials

The following supporting information can be downloaded at https://www.mdpi.com/article/10.3390/toxics13110926/s1, Table S1: Shapiro-Wilk test evaluation of measurable data title.
