# Supplementary material for: Assessment of Microplastic Exposure in Diabetic Patients Using Insulin
Source: Toxics. 2025 Oct 29;13(11):926. doi: 10.3390/toxics13110926 (PMC12656115; doi:10.3390/toxics13110926)
Supplement: Supplementary file 1 [file toxics-13-00926-s001.zip › toxics-3935824-supplementary.pdf]

Supplementary Table S1: Shapiro-Wilk test evaluation of measurable data title.

**Variable**	**Shapiro–Wilk W**	***p*-value**	**Normality**	**Test Used**
Age	0.976	0.123	Normal	Independent t-test
BMI	0.983	0.172	Normal	Independent t-test
HbA1c (%)	0.891	<0.001	Non-normal	Mann–Whitney U
Glucose (mg/dL)	0.902	<0.001	Non-normal	Mann–Whitney U
Microplastic level (µg/mL)	0.915	<0.001	Non-normal	Mann–Whitney U
Duration of disease (years)	0.944	0.004	Non-normal	Kruskal–Wallis
